# Metastatic and aggressive renal cell carcinoma mimicking a unilateral choroidal tuberculoma

**DOI:** 10.5935/0004-2749.20230016

**Published:** 2023

**Authors:** Laís Mesquita Caetano, Jéssica Cararo Frossard, Fábio P. Saraiva, Luiz Guilherme Marchesi Mello, Thiago Cabral

**Affiliations:** 1 Departament of Specialized Medicine, Universidade Federal do Espírito Santo, Vitória, ES, Brazil.; 2 Vision Unit Center, Hospital Universitário Cassiano Antonio de Moraes, Universidade Federal do Espírito Santo, Vitória, ES, Brazil.; 3 Clinical Division of Ophthalmology, Universidade de São Paulo, São Paulo, SP, Brazil.; 4 Departament of Ophthalmology, Universidade de São Paulo, São Paulo, SP, Brazil.

**Keywords:** Kidney neoplasms/complications, Metastases, neoplasm, Carcinoma, renal cell, Choroid neoplasms/etiology, Case reports, Neoplasias renais/complicações, Metástase neoplásica, Carcinoma de células renais, Neoplasias da coroide/ etiologia, Humanos, Relatos de casos

## Abstract

Ocular metastases from systemic tumors are uncommon. The choroid is the most frequent target, with a preference for elderly individuals. Lung cancer is the predominant primary tumor that metastasizes to the eyes in males, although other ocular conditions such as uveitis and retinal lesions can mimic secondary tumor implants in ocular tissues. On fundoscopy, choroidal metastasis resembles other infectious processes, especially choroidal tuberculoma. Therefore, patients presenting with choroidal masses should undergo detailed clinical examinations, especially if the mass is the first manifestation of a systemic and severe disease. In this report, we describe a young man with a metastatic choroidal tumor secondary to papillary renal cell carcinoma mimicking a unilateral choroidal tuberculoma.

## Abstract (pt)

A disseminação metastática ocular de tumores sistêmicos é incomum, ocorrendo principalmente na coroide e em pacientes idosos. O câncer de pulmão é considerado o principal tumor metastático ocular em homens, contudo, outras doenças oculares, como as uveítes e lesões retinianas, podem mimetizar os implantes secundários tumorais nos tecidos oculares. O aspecto fundoscópico das neoplasias da coroide pode apresentar similaridade com outros processos infecciosos, especialmente o tuberculoma de coroide. Dessa forma, a investigação clínica detalhada é de grande importância no diagnóstico de pacientes com massas coroideanas, especialmente quando configuram a primeira manifestação de uma doença sistêmica e grave. Relatamos um caso raro de metástase coroideana como primeira manifestação clínica do carcinoma de células renais em um homem jovem, mimetizando um tuberculoma de coroide.

## INTRODUCTION

Tuberculosis (TB) is an airborne infection caused by *Mycobacterium tuberculosis* (MTB), typically inducing the formation of granulomas in the lungs and, occasionally, in extrapulmonary tissues. Ocular involvement is observed in 1% of patients with pulmonary TB. The diagnosis of ocular TB can be challenging and relies on five pillars as follows: i) clinical condition compatible with ocular TB (especially in the presence of inflammation), ii) microbiological confirmation of intraocular MTB, iii) systemic evidence of TB (positive skin or serum tests and compatible pulmonary or extrapulmonary lesions), iv) ruling out other possible causes, and v) positivity in the MTB purified protein derivative (PPD) test within 4-6 weeks^(1)^. Less commonly, ocular TB may present as choroidal tuberculoma (a yellowish subretinal choroidal mass), usually in association with exudative retinal detachment and mimicking an infectious abscess or ocular tumor^(2)^.

Most malignant ocular tumors are uveal metastases, of which nearly 90% affect the choroid. The most frequent primary sites are the breasts (47%), lungs (21%), and gastrointestinal tract (4%), but in almost 15% of cases, the primary site is unknown at the time of diagnosis. Most choroidal metastases have an unspecific presentation, including a single yellowish lesion, usually in the posterior pole, associated with subretinal fluid^(3)^. Ultrasonography (US) is an important modality to aid in the differential diagnosis of intraocular lesions. Uveal metastases display medium-to-high internal echogenicity and smaller height-to-base ratios than intraocular melanomas^(4)^, but metastases from primary tumors can be difficult to distinguish from lesions secondary to trauma or infections such as choroidal tuberculoma.

In this case report, we describe a rare case of choroidal metastasis from renal cell carcinoma (RCC) in a young male patient with tuberculosis and highlight the importance of detailed and timely investigations of neoplasia in ocular masses.

## CASE REPORT

A 44-year-old black Brazilian male complained of pain in the left eye (oculus sinister [OS]) for 20 days, with no relation to eye movement but with association with reduced visual acuity. The patient reported having lost 10 kg in 1 month and a dry cough in the preceding 2 weeks. No other symptoms were reported, and no ocular, systemic, and familial antecedents were identified.

**Figure 1:**
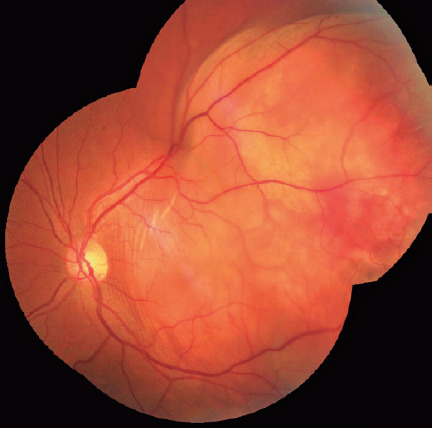
Mosaic image of retinography of the left eye, showing a whiteyellowish lesion in the choroid and/or deep retina in the posterior pole and superotemporal periphery, with mild subretinal hemorrhage and perilesional retinal detachment.

The ophthalmologic evaluation revealed a best-corrected visual acuity of 20/20 in the right eye (oculus dextrus [OD]) and counting fingers at 1.5 m in the left eye (OS). No changes were found on the biomicroscopy of the anterior segment in either eye (oculus uterque [OU]), including no anterior chamber or vitreous reaction. The intraocular pressure was 12 mmHg in OU. Fundoscopy revealed no abnormalities in the OD but revealed a large yellowish choroidal lesion in the temporal region of the posterior pole of the OS, with mild subretinal hemorrhage, associated with serous detachment of the perilesional retina (Figure 1A). US of the OS confirmed the presence of a choroidal mass temporally to the fovea, with medium and homogenous internal echogenicity, associated with serous retinal detachment (Figure 1B).

**Figure 2:**
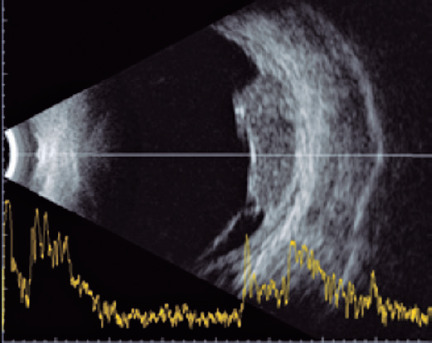
Ultrasonography image of the left eye, showing a choroidal mass temporally to the fovea, with medium and homogenous internal echogenicity and serous detachment of the perilesional retina.

The results of the serological studies for syphilis, toxoplasmosis, and human immunodeficiency virus were negative, and the PPD test results was positive (10 mm of induration). Chest computed tomography (CT) revealed i) multiple nodules with soft tissue density throughout the lung parenchyma, especially on the right side (Figure 2A); ii) lymph node enlargement in the right paratracheal and hilar chains and carinal chain, with enhancement suggestive of necrosis; and iii) a small cortical Bosniak category I cyst in the right kidney (Figure 2B). Bronchoscopy with lung biopsy revealed signs of anthracosis and unspecific inflammation, but the bronchoalveolar lavage was positive (3+) on bacilloscopy for MTB. On the basis of the diagnostic suspicion of disseminated systemic TB, the patient was referred to an infectologist and introduced to rifampicin, isoniazid, pyrazinamide, and ethambutol (RIPE) therapy.

**Figure 3:**
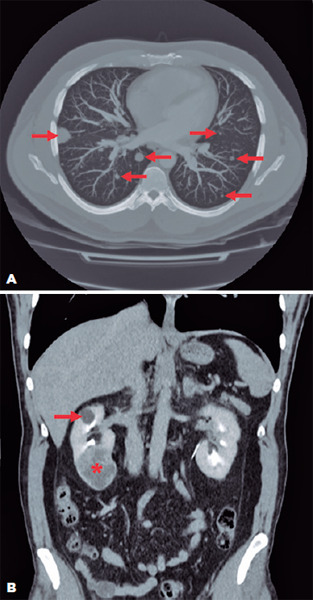
(A) Chest computed tomography (CT) image showing multiple nodules with a soft tissue density dispersed across the lung parenchyma (red arrows). (B) Abdominal CT image showing a small cortical Bosniak category I cyst in the right kidney (red arrow) and a partially exophytic expansive nodular lesion in the lower cortex of the right kidney, with a probable neoplastic etiology (red asterisk).

However, the lesion worsened even after anti-tuberculin treatment. An extended evaluation of the renal cyst, performed with abdominal CT, revealed i) multiple hepatic and adrenal glands nodules, and ii) a partially exophytic expansive nodular lesion in the lower cortex of the right kidney, with heterogeneous contrast enhancement and central areas of necrosis/liquefaction, with a probable neoplastic etiology (Figure 2B). The liver nodule fragments (Figure 3) obtained using transcutaneous biopsy were submitted for immunohistochemistry for vimentin, PAX-8, and cytokeratin 7 (all positive) and cytokeratin 20 and thyroid transcription factor 1 (both negative), confirming the diagnosis of papillary RCC. A diagnostic vitrectomy was scheduled, but the patient died of respiratory failure 16 days after the diagnosis of RCC.

## DISCUSSION

Ocular metastases are uncommon and generally target the choroid. The prevalence of neoplastic dissemination to ocular tissues depends mostly on the primary site and the patient’s sex^(3)^. Ocular metastases are predominantly from lung carcinoma in male patients and from breast adenocarcinoma in women^(5)^. RCC accounts for 1-3% of all malignant visceral neoplasms, with a preference for the male sex, and is associated with high mortality rates^(6)^. Representing only 10% of all cases, papillary RCC is the least prevalent subtypes^(7)^. The classic triad of clinical symptoms includes flank pain, gross hematuria, and palpable renal mass but is present in only 10% of all cases at the time of diagnosis (an indication of advanced disease)^(7)^. RCC metastasizes at an early stage, targeting mainly the lungs, regional lymph nodes, bones, liver, and brain^(8)^. According to a study, RCC is responsible for only 3.57% of all ocular and orbital metastases^(9)^. However, the fact that nearly 90% of patients with ocular tumors present no systemic symptoms at the time of diagnosis justifies the inclusion of metastatic RCC in the differential diagnosis^(9)^.

In the present case, the diagnosis was confounded by several circumstances as follows: i) ocular metastasis of RCC is rare; ii) the ocular symptoms were practically the only manifestations of the disease; iii) tuberculosis is highly prevalent in Brazil (considered one of the 20 countries with the highest estimated number of incident TB cases in the world)^(10)^; iv) choroidal lesions tend to be unspecific; and v) the patient condition mimicked choroidal tuberculoma. The patient was examined for the classic five pillars of TB (clinical condition compatible with ocular TB; microbiological confirmation of intraocular MTB; systemic evidence of TB; ruling out of other causes; positivity in the tuberculin skin test)^(1)^ but showed no signs of inflammation in the anterior chamber or vitreous, a typical finding in immunocompromised subjects with ocular infections. Moreover, the pulmonary images were not typical of TB, and the ocular lesion worsened even after anti-tuberculin treatment. Diagnostic vitrectomy, with cytopathological analysis to identify tumor cells or polymerase chain reaction analysis for MTB, was not possible because of the patient’s untimely death. However, the absence of intraocular inflammation during the entire follow-up, the failure of the RIPE regimen to reduce the lesion, and the presence of metastatic RCC led to the diagnosis of metastatic choroidal tumor secondary to papillary RCC.

The etiology of intraocular tumoral lesions can be difficult to establish. Uveitis and neoplasms are the main differential diagnoses. The endemicity of certain infectious diseases can lead to a misdiagnosis of uveitis if the case is not thoroughly investigated. RCC is not a common tumor and rarely involves the eyes. Diagnosis is made more difficult by the frequent absence of systemic signs and symptoms at the time of ocular manifestation. Ophthalmologists are advised to conduct a detailed clinical investigation (including laboratory tests and imaging) of patients with intraocular masses. In this report, we describe a young man with a bacilloscopy result positive for MTB who presented a metastatic choroidal tumor secondary to papillary RCC initially mimicking a unilateral choroidal tuberculoma.

## Figures and Tables

**Figure 4:**
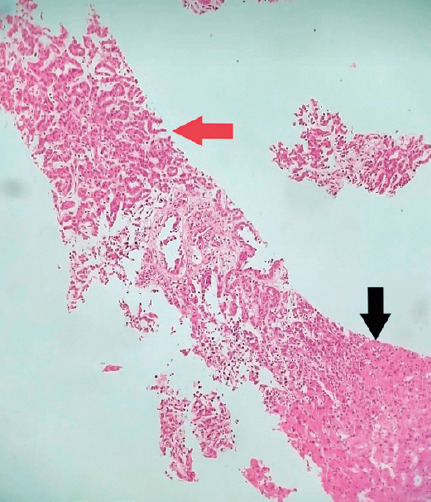
Histological slide of a hepatic nodule fragment stained with hematoxylin and eosin in 20-fold magnification, showing an atypical cell proliferation forming microtubular arrangements (red arrow) infiltrating the hepatic stroma (black arrow).
